# MiRNA-29b and miRNA-497 Modulate the Expression of Carboxypeptidase X Member 2, a Candidate Gene Associated with Left Ventricular Hypertrophy

**DOI:** 10.3390/ijms23042263

**Published:** 2022-02-18

**Authors:** Jana Subrova, Karen Böhme, Allan Gillespie, Miriam Orphal, Claudia Plum, Reinhold Kreutz, Andreas Eisenreich

**Affiliations:** Institute of Clinical Pharmacology and Toxicology, Charité—Universitätsmedizin Berlin, Corporate Member of Freie Universität Berlin, Humboldt-Universität zu Berlin, and Berlin Institute of Health, 10117 Berlin, Germany; karen.boehme@charite.de (K.B.); allan.gillespie@charite.de (A.G.); miriam.orphal@charite.de (M.O.); claudia.plum@charite.de (C.P.); reinhold.kreutz@charite.de (R.K.); andreas.eisenreich@gmx.de (A.E.)

**Keywords:** carboxypeptidase X member 2, microRNA, posttranscriptional, gene expression, left ventricular hypertrophy

## Abstract

Left ventricular hypertrophy (LVH) is a major risk factor for adverse cardiovascular events. Recently, a novel candidate gene encoding the carboxypeptidase X member 2 (CPXM2) was found to be associated with hypertension-induced LVH. CPXM2 belongs to the M14 family of metallocarboxypeptidases, yet it lacks detectable enzyme activity, and its function remains unknown. Here, we investigated the impact of micro (mi)RNA-29b, miRNA-195, and miRNA-497 on the posttranscriptional expression control of CPXM2. Candidate miRNAs for CPXM2 expression control were identified in silico. CPXM2 expression in rat cardiomyocytes (H9C2) was characterized via real-time PCR, Western blotting, and immunofluorescence. Direct miRNA/target mRNA interaction was analysed by dual luciferase assay. CPXM2 was expressed in H9C2 and co-localised with z-disc associated protein PDZ and LIM domain 3 (Pdlim3). Transfection of H9C2 with miRNA-29b, miRNA-195, and miRNA-497 led to decreased levels of CPXM2 mRNA and protein, respectively. Results of dual luciferase assays revealed that miRNA-29b and miRNA-497, but not miRNA-195, directly regulated CPXM2 expression on a posttranscriptional level via binding to the 3′UTR of CPXM2 mRNA. We identified two miRNAs capable of the direct posttranscriptional expression control of CPXM2 expression in rat cardiomyocytes. This novel data may help to shed more light on the—so far—widely unexplored expression control of CPXM2 and its potential role in LVH.

## 1. Introduction

Cardiovascular diseases (CVDs) remain the most prevalent cause of morbidity and mortality worldwide [1,2,3]. High blood pressure is one of the principal risk factors for the development of CVDs, such as heart failure, coronary heart disease, atrial fibrillation, and stroke [4]. Although intensive research on the field of pathogenesis in cardiovascular system has been performed for decades, molecular mechanisms leading to CVDs in humans are still not entirely understood [5]. Yet, the knowledge about the molecular pathways involved in the development and progression of CVDs is essential in the search for more efficient targeted therapies.

Carboxypeptidase (CP) X member 2 (CPXM2) is a member of the M14 family and the N/E subfamily of metallocarboxypeptidases [6]. However, it does not show any detectable enzymatic activity. Recently, the gene encoding CPXM2 was found to be associated with the development of hypertension-induced left ventricular hypertrophy (LVH) in mice [7]. CPXM2 overexpression was also reported to promote proliferation, migration and the epithelial to mesenchymal transition of osteosarcoma cells [8], as well as gastric cancer cells [9].

CPs represent a large group of enzymes involved in the C-terminal cleavage of different substrates, such as food proteins and neuropeptides [10]. The N/E subfamily contains three members which lack catalytic function. In addition to CPXM2, CP X member 1 (CPXM1) and aortic CP-like protein/adipocyte enhancer-binding protein 1 (ACLP/AEBP1) were found not to hydrolyse any of the tested CP substrates [6]. These findings are consistent with the fact that the CP domains of CPXM1, CPXM2, and AEBP1 lack different amino acid residues responsible for the catalytic function and/or substrate binding in active CPs [11].

CPXM2 was first discovered and described by Xin et al. in 1998. CPXM2 is expressed in various tissues, including brain, liver [6], inner ear [12] and heart [7]. Moreover, its sequence is evolutionarily conserved in different species, such as zebrafish, mice and humans (https://www.ncbi.nlm.nih.gov/gene/?term=cpxm2 (accessed on 14 January 2022)). This indicates that CPXM2 may play an important biological role. However, its distinct (patho-)physiological function is widely unknown.

**Figure 1:**
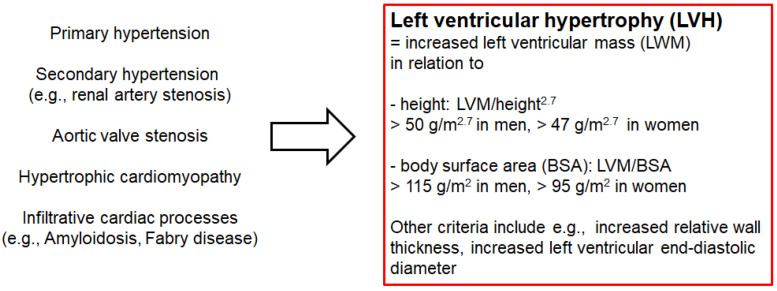
Different (patho-)physiologic conditions that can lead to left ventricular hypertrophy (LVH) in humans. Various CVDs as well as athletic training are associated with LVH. According to the American Society of Echocardiography and the European Association of Cardiovascular Imaging, LVH is defined as an increased left ventricular mass (LVM) in relation with height or body surface area. LVH can be determined via echocardiography or cardiac magnetic resonance imaging.

High blood pressure is one of the most common causes of heart hypertrophy [13,14,15]. Additionally, LVH can result from athletic training [16] associated with physiologic higher plasma cardiac biomarkers, such as troponin I [17]. On the other hand, increased heart-wall thickness can also develop independent from high blood pressure due to primary heart diseases, e.g., hypertrophic cardiomyopathy [18] or cardiac amyloidosis [19,20]. The most prevalent causes of LVH are summarized in Figure 1. Mechanical stress evokes altered expression patterns in cardiac myocytes [21]. Various z-disc-associated proteins are involved in this process called mechanotransduction, such as PDZ and LIM domain 3 (Pdlim3; PDZ stands for postsynaptic density 95, discs large, and zonula occludens-1; LIM was originally described in the proteins LIN-11, Isl1m, and MEC-3), also known as actinin-associated LIM protein (ALP) [22].

Micro (mi)RNAs are short (~22 nucleotides long), single-stranded, non-coding RNAs [23,24]. They act as posttranscriptional regulators of gene expression via binding to regulatory sequences in the 3′-untranslated region (3′UTR) of their corresponding target messenger (m)RNAs, thus leading to either repression of the translation or degradation of the target mRNA [25]. Various relevant processes involved in the development of CVDs, such as proliferation, apoptosis, inflammation, and fibrosis are influenced by miRNAs [26,27,28,29,30,31]. For example, miRNA (miR)-29b attenuated organ fibrosis in the cardiovascular system, and other tissues [32,33,34,35,36] via inhibiting the expression of collagens and other extracellular matrix proteins [37]. Members of the miR-15 family were also identified to be involved in cardiac diseases [38,39,40]. Amongst other things, miR-15 family members mediated a protective effect against heart hypertrophy and fibrosis via the repression of the transforming growth factor β (TGFβ) pathway [41]. Moreover, miRNAs themselves can be downregulated by long non-coding RNAs (lncRNAs) [42]. Interactions on the lncRNA-miRNA axis seem to play an important role in the pathophysiology of CVDs and may imply novel targeted therapies in this field [43,44,45]. Additionally, miRNAs can serve as useful diagnostic markers for various diseases [46,47,48,49].

In this study, we identified miRNA candidates able to regulate the expression of CPXM2 on a posttranscriptional level. Here, we showed a significant reduction of CPXM2 mRNA and protein in rat cardiomyocytes after transfection with miRNA-29b, miRNA-497, and miRNA-195, respectively. With a dual luciferase reporter assay, we were able to confirm a direct interaction between miRNA-29b, and miRNA-497, respectively, with their in silico-predicted binding sides in the 3′UTR of the CPXM2 mRNA. Our data provide new insights into the expression control of CPXM2 in the context of its potential functional role in cardiovascular biology.

## 2. Results

### 2.1. CPXM2 Is Expressed in Rat Cardiac Cells

**Figure 2:**
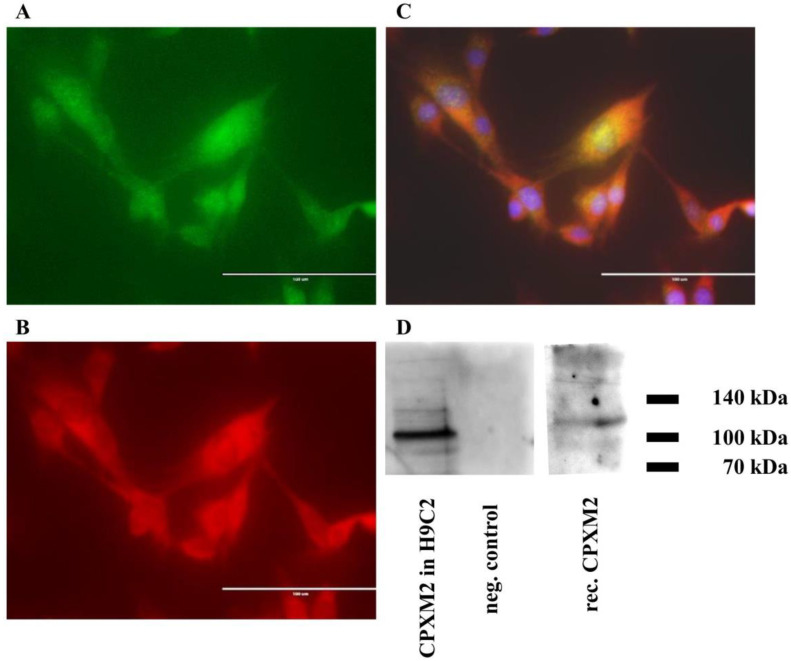
Expression of CPXM2 and Pdlim3 in rat cardiomyocytes. H9C2 cells were fixed and double-labelled for confocal indirect immunofluorescence microscopy with the polyclonal (**A**) anti-CPXM2 and (**B**) anti-Pdlim3 antibodies. In the overlay image (**C**) intracellular co-localisation of CPXM2 and Pdlim3 in rat cardiomyocytes is depicted. Nuclear DNA was stained with DAPI (shown in blue). In (**D**) the CPXM2 protein expression in H9C2 as well as a positive control (human recombinant CPXM2) and negative control (empty well) are shown. Bar = 100 μm.

Immunofluorescence analysis revealed that CPXM2 was expressed in H9C2 rat cardiomyocytes (Figure 2A–C). In H9C2 cells, CPXM2 was co-localized with the z-disc-associated protein Pdlim3. Western blot analysis confirmed the presence of CPXM2 protein in H9C2 (Figure 2D).

### 2.2. Identification of miR-29b, mir-195, and miR-497 as Candidates for Posttranscriptional CPXM2 Expression Control

**Figure 3:**
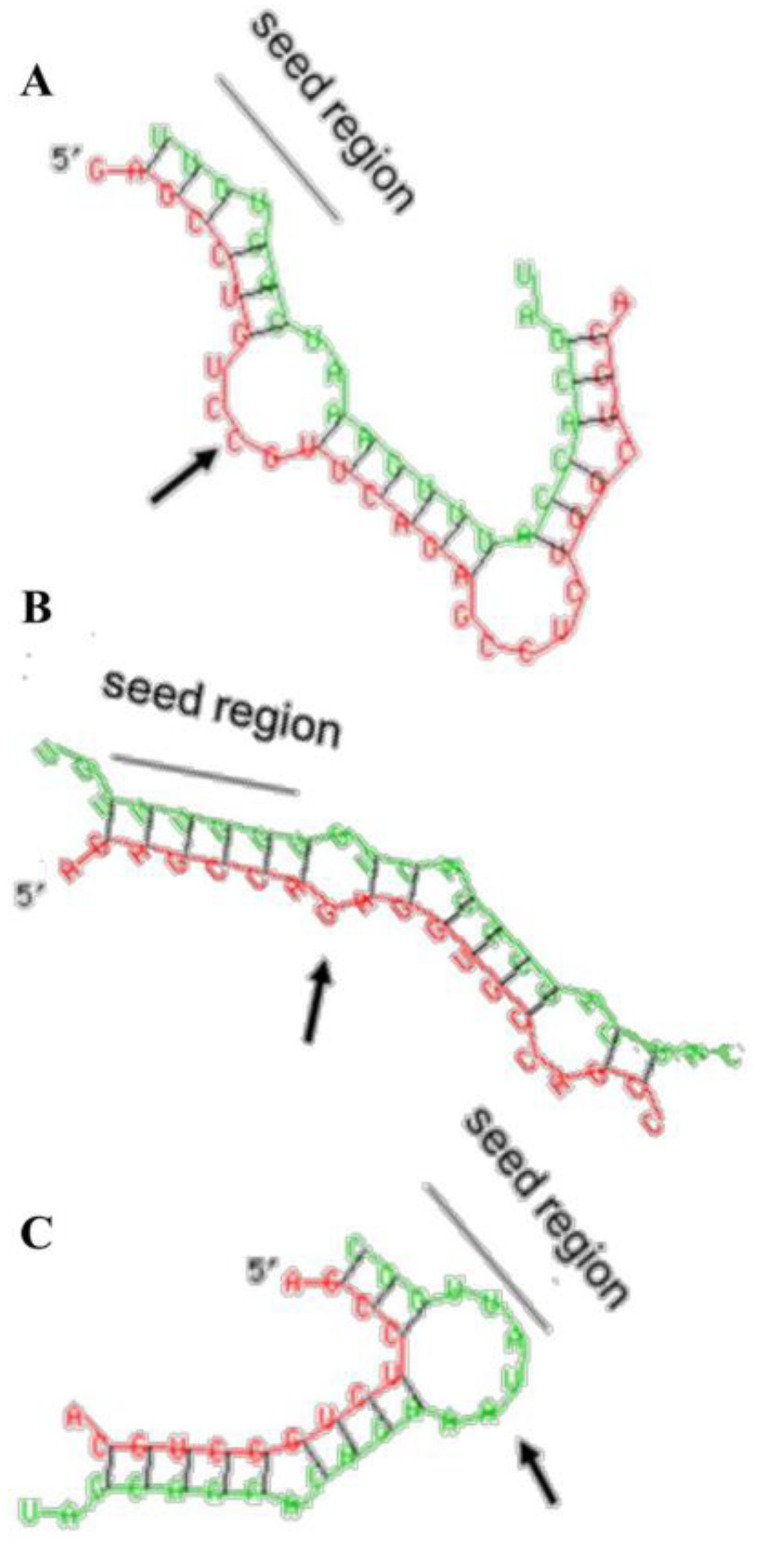
Structural in silico analysis of the miRNA/target mRNA interaction. Computational models of miRNA/mRNA interaction between (**A**) miR-29b, (**B**) miR-497, and (**C**) miR-195, respectively, with a corresponding sequence in the 3′UTR of CPXM2 mRNA. (green) miRNA; (red) CPXM2 mRNA 3′UTR sequence; (seed region) miRNA nucleotides 2–8; (arrow) central bulge.

Using several independent miRNA databases, approximately 20 different miRNAs were predicted to potentially bind to the 3′UTR of CPXM2 (data not shown). For three of them, miR-29b, miR-195, and miR-497, structural in silico analysis of the miRNA/target mRNA interaction indicated a possible formation of regulatory or stabilizing structures [24]. MiR-29b and mit-497 showed two important attributes necessary for the building of a stable miRNA/target mRNA complex: a perfect complementarity of miRNA bases 2–8 (seed region) with the corresponding mRNA sequences and the existence of a central bulge in the miRNA/target mRNA complexes (Figure 3A,B). The miR-195/CPXM2 3′UTR duplex exhibited only the presence of a central bulge, but the seed region of miR-195 was not perfectly complementary to the corresponding mRNA sequence (Figure 3C).

### 2.3. MiR-29b, mir-195, and miR-497 Suppressed the Expression of CPXM2

**Figure 4:**
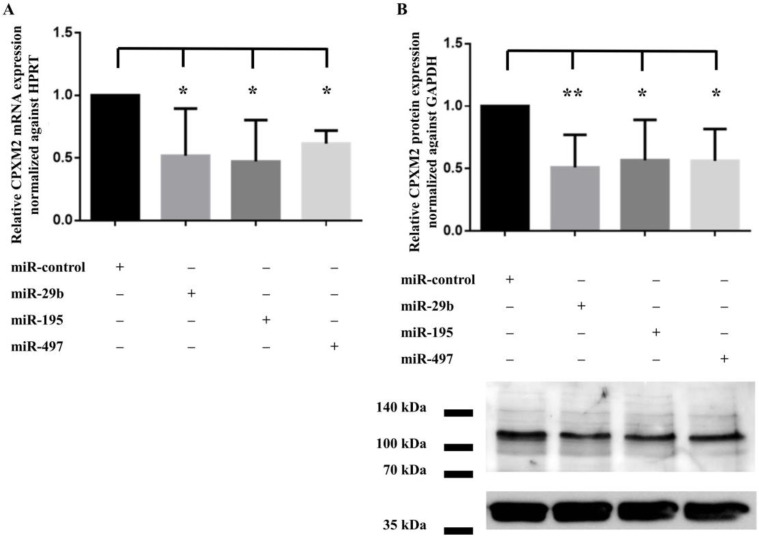
Impact of miR-29b, miR-195, and miR-497 on CPXM2 expression in H9C2 cells. Shown is the relative reduction of CPXM2 (**A**) mRNA and (**B**) protein expression in H9C2 48 h after transfection with miR-29b, miR-195, miR-497, or a negative control miRNA mimics (miR-control), respectively. CPXM2 mRNA expression was normalized against HPRT. CPXM2 protein expression was normalized against GAPDH. Data were presented as relative (x-fold) expression change. (*) *p* < 0.05; (**) *p* < 0.01; *n* ≥ 3.

To determine whether the miRNA candidates acted as posttranscriptional regulators of CPXM2 expression in cardiac cells, we transfected H9C2 with miR-29b, miR-195, miR-497, and negative control miRNA mimics, respectively. Treatment of cells with all three miRNA candidates significantly reduced the CPXM2 mRNA expression after 48 h (Figure 4A). A comparable effect was also found on protein level 48 h after transfection. Compared to the controls, CPXM2 protein expression was significantly reduced in H9C2 by miR-29b, miR-195, and miR-497, respectively (Figure 4B).

### 2.4. MiR-29b, and miR-497 Inhibit CPXM2 Expression through Direct Binding to Regulatory Sequences in the 3′UTR of CPXM2 mRNA

**Figure 5:**
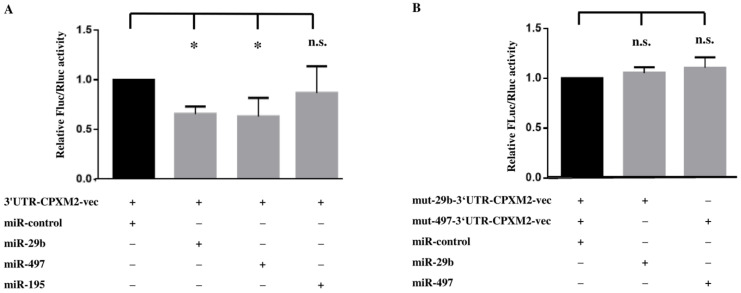
Dual luciferase reporter assay in H9C2. (**A**) Shown is the influence of miR-29b, miR-497, and miR-195, respectively, on the relative firefly luciferase activity of the miRNA 3′UTR target clone pEZX-MT06, containing the 3′UTR of CPXM2 (3′UTR-CPXM2-vec) 48 h after transfection. (**B**) Depicts the impact of miR-29b and miR-497 on the relative firefly luciferase activity, respectively, containing the 3′UTR of CPXM2 with mutated binding sides for miR-29b (mut-29b-3′UTR-CPXM2-vec), or miR-497 (mut-497-3′UTR-CPXM2-vec), respectively, 48 h after transfection. Firefly luciferase activity was normalized against renilla luciferase activity. (*) *p* < 0.05; (n.s.) no significant difference; *n* ≥ 3; FLuc, firefly luciferase activity; RLuc, renilla luciferase activity; miR-control, negative control miRNA mimics.

Dual luciferase reporter assays were performed to analyse whether the inhibitory impact of the miRNA candidates on CPXM2 was mediated by direct interaction with the 3′UTR of CPXM2 mRNA. Co-transfection of H9C2 with miR-29b or miR-497, respectively, and luciferase reporter vector containing the 3′UTR of CPXM2 led to a significant reduction of luciferase activity, compared to cells treated with negative control miRNA mimics (Figure 5A). For miR-195, no significant effect on luciferase activity was detected (Figure 5A).

To verify that the effect of candidate miRNAs on the luciferase activity was due to direct binding to the predicted sides in the CPXM2 3′UTR, we mutated the corresponding binding sides and repeated the experiment with the mutated vectors. Mutation of predicted binding sides completely abolished the inhibitory impact of miR-29b and miR-497 on luciferase activity in this experimental setting (Figure 5B).

## 3. Discussion

Although CPXM2 was first described more than 20 years ago [6], there is still little evidence about its expression control and biological role. In the present study, we determined its expression and localization in H9C2 rat cardiomyocytes, representing a relevant in vitro model. Moreover, we characterized the impact of three candidate miRNAs on the posttranscriptional control of CPXM2 expression in H9C2 cells. Concerning the evolutionary conservation of CPXM2 among different species, our findings should be applicable to human cardiomyocytes as well. To verify this, future studies should be carried out.

### 3.1. CPXM2 Is Expressed in Rat Cardiomyocytes and Co-Localized with Pdlim3

Immunofluorescence and Western blot analysis showed that CPXM2 was expressed in rat cardiomyocytes. This is consistent with the findings of Grabowski et al. who explored CPXM2 in the cardiac tissue of rats, mice, and humans [7,50]. Furthermore, we demonstrated the co-localisation of CPXM2 with Pdlim3 in H9C2 (Figure 2A–C). Pdlim3 is a z-disc protein which plays an important role in signal transduction, cell proliferation and cytoskeleton assembly in cardiac myocytes [51,52]. Lodder et al. associated elevated Pdlim3 levels with increased collagen deposition in the heart, a process involved in fibrosis in the context of cardiac disease [53]. Considering its co-localisation with Pdlim3, CPXM2 could potentially be involved in Pdlim3-mediated effects during the development of heart hypertrophy and fibrosis.

CPXM2, similar to two other related proteins sharing a high sequence homology with CPXM2 (CPXM1 and AEBP1) contains a discoidin domain, which is not present in any other metallocarboxypeptidase family member [10,11]. Discoidin motive can be found in many eukaryotic and prokaryotic proteins. It usually serves as a binding domain for different ligands, such as growth factors, galactose or collagens [54]. Both CPXM1 and AEBP1 are able to bind collagen [11,55]. Therefore, it is conceivable that CPXM2 may also interact with collagen, which—in turn—may affect cardiac fibrosis. However, further experiments are needed to test the ability of CPXM2 to bind collagen and to examine the possible involvement of CPXM2 in the process of cardiac fibrosis.

### 3.2. CPXM2 Expression Is Directly Regulated via miR-29b and via miR-497

MiRNAs influence many cellular functions via suppressing the generation of their corresponding target proteins on a posttranscriptional level [56]. MiRNAs have been associated with the pathogenesis of several cardiovascular pathologies, such as heart hypertrophy and fibrosis [36,57,58,59,60,61]. Here, we showed for the first time that miR-29b, as well as miR-497, reduced CPXM2 expression in H9C2 through its specific binding to the 3′UTR of CPXM2 mRNA.

Other groups reported elevated miRNA-29b levels to be associated with antifibrotic effects in the heart and other tissues [32,34,62,63,64,65]. For example, Monaghan et al. showed that local delivery of miR-29b into heart tissue shortly after myocardial infarction positively influenced postischemic cardiac remodelling in mice [32]. Zhang et al. demonstrated that miR-29b overexpression prevented angiotensin II-induced cardiac fibrosis by targeting the TGFβ pathway [65]. Qin et al. also found miR-29b to be involved in TGFβ signalling. MiR-29 reduced TGFβ-mediated renal fibrosis in mice with obstructive nephropathy [34].

The other miRNA candidate from this study, miR-497, was also shown to play an important role in the development of CVDs in previous studies [39,60]. Xiao et al. described in 2016 that treatment of primary mouse cardiomyocytes with angiotensin II led to lowered miR-497 levels in an in vitro model of cardiac hypertrophy [60]. Moreover, miR-497 overexpression led to a decrease in various hypertrophy markers, such as cell area and atrial natriuretic peptides.

**Figure 6:**
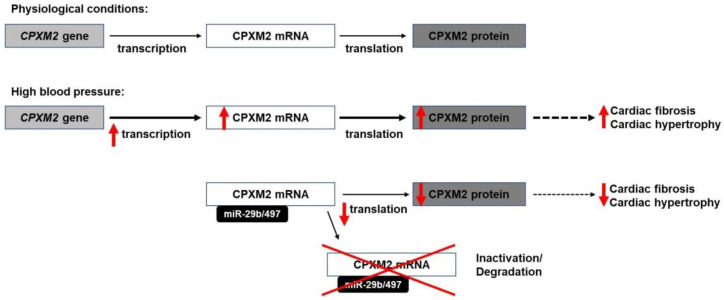
Possible effects of CPXM2 expression regulation via miRNA-29b and miRNA-497. According to Grabowski at al., CPXM2 is overexpressed under high blood pressure conditions. miRNA-mediated CPXM2 downregulation may reduce cardiac hypertrophy and fibrosis. Red arrow indicates an increase (↑) or a decrease (↓).

Together, these studies indicate a potential role of miR-29b and miR-497 in fibrosis, cellular remodelling, and in the development of heart hypertrophy, which may be potentially mediated via modulating CPXM2 expression (Figure 6). However, further studies should be performed to explore the molecular pathways in which CPXM2 may be involved to better understand its biological function. Deeper knowledge about the pathophysiological role of CPXM2 in the development of cardiac hypertrophy and fibrosis could help us to implement novel targeted therapies for these conditions. MiRNAs, in general, have been shown to act as potential therapeutic agents [66,67]. For example, Ban et al. demonstrated miRNA-497 to accelerate wound healing in diabetic mice after local injection [68]. Thus, our findings could contribute to the development of future miRNA-based therapeutics for hypertension-induced CVDs.

In addition to the direct effects of miR-29b and miR-497, we also found a significant inhibitory effect of miR-195 on the CPXM2 expression in H9C2. However, in contrast to the other tested miRNA candidates, miR-195 did not reduce luciferase activity, indicating no direct interaction of miR-195 with the CPXM2 mRNA (Figure 5A). This finding is in line with the lack of perfect base pairing between the seed region of miR-195 and the corresponding sequence in the CPXM2 3′UTR revealed by our in silico analysis (Figure 3C). Possibly, miR-195 reduced the CPXM2 expression via indirect effects. Zhang et al. show miR-195 to be involved in the TGFβ signalling pathway, thus attenuating cardiac hypertrophy [69]. It may be possible that miR-195 influences the CPXM2 expression via the TGFβ signalling pathway. However, further research would be needed to verify this hypothesis.

In addition, more experiments should be carried out to better understand the role of CPXM2 in cardiac tissue. Until now, we were not able to show a functional influence of miRNA-induced CPXM2 downregulation on cell signalling in H9C2 cells. Our pilot studies (data not shown) did not reveal a significant impact of the tested miRNA candidates via the transfection of H9C2 on the phosphorylation state of factors involved in the phosphatidylinositol 3-kinase/protein kinase B/mammalian target of rapamycin (PI3K/Akt/mTOR) pathway, which could indicate a potential influence of CPXM2 on cell survival and proliferation [70]. Further research is necessary to examine the involvement of CPXM2 on cell signalling or the other biological effects of CPXM2 in cardiomyocytes. Such analyses are planned for future studies.

## 4. Materials and Methods

### 4.1. Cell Culture

Rat cardiomyocytes (H9C2) were cultured in Dulbecco’s Modified Eagle Medium (DMEM) with 10% foetal bovine serum (FBS), and 1% penicillin/streptomycin, all purchased from Biochrom GmbH, Berlin, Germany. H9C2 were kept at 37 °C and in a humidified 5% CO_2_ atmosphere. Before transfection, cells were starved with FBS-free DMEM for at least 12 h. Transfection of H9C2 was performed using 200 nM miRNA-mimics for miR-29b, miR-195, miR-497, and nonsense miRNA control (miR-control), all provided by Sigma-Aldrich Chemie GmbH by Merck, Munich, Germany. Lipofectamine^®^2000 was supplied by Thermo Fisher Scientific, Carlsbad, CA, USA. Transfection efficiency, tested via fluorescence-activated cell sorting 6 h after transfection with 200 nM Dy547 transfection control (Dharmacon, Lafayette, CO, USA), was 62% in H9C2.

### 4.2. In Silico Analysis

Potential miRNA candidates capable of regulating the CPXM2 expression were identified using the miRNA databases TargetScan (http://www.TargetScan.org), miRDB (http://miRDB.org), and microRNA.org—Targets and Expression (http://www.microRNA.org), all accessed on 18 April 2016. Structural in silico analysis of the miRNA/mRNA interaction was performed using the RNAhybrid 2.2 database (https://bibiserv.cebitec.uni-bielefeld.de/rnahybrid (accessed on 20 April 2016)).

### 4.3. Immunofluorescence Staining

For immunofluorescence analysis, H9C2 cells were fixated with 4% formaldehyde and permeabilized with 0.5% Triton X-100 for 10 min. Three washing steps with 1× phosphate-buffered saline (PBS) were performed to remove residual formaldehyde. To prevent unspecific binding, cells were blocked for 20 min at room temperature in PBS with 5% FBS and 0.1% Triton X100. Samples were then directly incubated with polyclonal rabbit anti-rat CPXM2 antibodies (Thermo Fisher, Carlsbad, CA, USA) 1:100 for 1 h at room temperature. For detection, after three washings steps in 1× PBS, fluorescein isothiocyanate (FITC)-labelled goat anti-rabbit antibodies (#12-507, Millipore by Merck, Darmstadt, Germany) were used in a 1:200 dilution for 1 h at room temperature in the dark. Next, after two washings steps in 1× PBS, samples were incubated with polyclonal ALEXA FLUOR^®^ 594 conjugated rabbit anti-rat Pdlim3 antibodies (#bs-2928R-A594, Bioss, Woburn, MA, USA) in a 1:200 dilution for 1 h at room temperature in the dark. Nuclear DNA was stained with 4′,6-Diamidino-2-Phenylindole (DAPI, #D1306, Thermo Fisher, Carlsbad, CA, USA), 1:5000, for 10 min at room temperature in the dark. Immunofluorescence was detected with a confocal microscope (Leica TCS SPE, Wetzlar, Germany) and analysed by the EVOS^®^ FL Auto Imaging System (Thermo Fisher, Carlsbad, CA, USA).

### 4.4. Quantitative Real-Time Polymerase Chain Reaction (RT-qPCR)

Total RNA was isolated 48 h after transfection via the universal RNA purification kit (Roboklon GmbH, #E3598-02, Berlin, Germany). Then, 1 μg of isolated RNA was reverse transcribed using the high-capacity cDNA reverse transcription kit (Thermo Fisher Scientific, #4368814, Carlsbad, CA, USA) under conditions: 65 °C, 5 min; 25 °C, 10 min; 37 °C, 120 min; 85 °C, 5 min. Subsequent, RT-qPCR was performed by using a 7500 Fast Real-Time PCR System (Thermo Fisher Scientific, Carlsbad, CA, USA) and the Fast SYBR^®^ Green Master Mix (Applied Biosystems, Darmstadt, Germany) for measurement of CPXM2 and hypoxanthine phosphoribosyltransferase (HPRT), following the manufacturer’s protocol. Primers CPXM2-f/CPXM2-r (CATCCCTGAGTGGTTTCTGTCTG/TGCTACAACCAGCTCACCCC) and HPRT-f/HPRT-r (CTCATGGACTGATTATGGACAGGACT/TCCAGCAGGTCAGCAAAGAAC) were used. PCR temperature conditions: 95 °C, 20 s; 50 cycles 95 °C, 3 s; 60 °C, 30 s. Relative quantification was performed using the ΔΔ-cq method [71].

### 4.5. Western Blotting

Proteins were isolated from H9C2 48 h after transfection. Western blot analyses of protein samples were carried out as described before [72]. In brief, protein samples were separated by sodium dodecyl sulfate polyacrylamide gel electrophoresis (SDS-PAGE) and transferred to polyvinylidene difluoride (PVDF) membrane (Carl Roth GmbH, Karlsruhe, Germany). For detection, specific rabbit anti-rat antibodies against CPXM2 (Thermo Fisher, Carlsbad, CA, USA) 1:300, and mouse anti-rat antibodies against glyceraldehyde 3-phosphate dehydrogenase (GAPDH, Calbiochem by Merck, Darmstadt, Germany) 1:500 were used (incubation overnight at 4 °C). Subsequently, the samples were incubated with corresponding secondary goat anti-rabbit antibodies (Sc-2004, Sigma-Aldrich Chemie GmbH by Merck, Munich, Germany) in a 1:5000 dilution and goat anti-mouse antibodies (# A4416-1ML, Sigma-Aldrich Chemie GmbH by Merck, Munich, Germany) in a 1:10000 dilution for 1 h at room temperature in the dark. Human recombinant CPXM2 protein (Abnova, Taipei, Taiwan) was used as a positive control. Blots were visualized and quantified by using FUSION FX7 (Peqlab Biotechnologie GmbH, Erlangen, Deutschland). Western blot results were quantified via Gel-Pro Analyzer software version 4.0.00.001 (Media Cybernetics, Bethesda, MD, USA).

### 4.6. Dual Luciferase Reporter Assay

Dual luciferase reporter assays were preformed using a miRNA 3′UTR target clone pEZX-MT06, containing the 3′UTR of CPXM2 (# HmiT002050-MT06) and the negative control vector (# CmiT000001-MT01), both supplied by GeneCopoeia, Rockville, MD, USA. Furthermore, two 3′UTR clones with a mutated binding side for miR-29b, or miR-497, respectively, were prepared using the Q5^®^ Site-Directed Mutagenesis Kit (New England BioLabs, Ipswich, MA, USA) according to the manufacturer’s protocol. H9C2 were co-transfected with 200 nM miR-29b, miR-195, or miR-497 mimics, and 200 ng of the luciferase reporter vector (wild type or mutated vectors, respectively) or the empty control vector in 96-well plates in total volume 100 μL of DMEM. Then, 48 h after transfection the luciferase reporter assay (Dual-Glo^®^ Luciferase Assay System, Promega, Madison, WI, USA) was performed, following the manufacturer’s instructions.

### 4.7. Statistical Analysis

All data were expressed as mean ± SEM. Data were analysed by Student’s *t*-test or one-way ANOVA, as appropriate. Statistical analyses were performed using GraphPad Prism v4.03 (GraphPad Software, Inc., La Jolla, CA, USA). A probability value (*p*) ≤ 0.05 was regarded as significant.

## 5. Conclusions

In summary, we showed for the first time that CPXM2 is expressed in rat cardiomyocytic cells. Moreover, we demonstrated miR-29b and miR-497 to regulate CPXM2 expression on a posttranscriptional level. This was mediated via the direct interaction of these miRNAs with the regulatory elements within the 3′UTR of CPXM2 mRNA. These novel findings may help to shed more light on the—so far—widely unexplored expression control of CPXM2 and may help to further characterize the functional role of this factor in the context of arterial hypertension-induced heart hypertrophy and cardiac fibrosis. Understanding of the pathophysiological function of CPXM2 in cardiac tissue could lead to development of future targeted therapies for CVDs.

## Data Availability

The datasets generated and/or analysed during the current study are available from the corresponding author on reasonable request.
